# Puerarin Inhibits NLRP3-Caspase-1-GSDMD-Mediated Pyroptosis via P2X7 Receptor in Cardiomyocytes and Macrophages

**DOI:** 10.3390/ijms241713169

**Published:** 2023-08-24

**Authors:** Shuchan Sun, Difei Gong, Ruiqi Liu, Ranran Wang, Di Chen, Tianyi Yuan, Shoubao Wang, Cheng Xing, Yang Lv, Guanhua Du, Lianhua Fang

**Affiliations:** 1 State Key Laboratory of Bioactive Substances and Functions of Natural Medicines, Institute of Materia Medica, Chinese Academy of Medical Sciences and Peking Union Medical College, Beijing 100050, China; shuchansun@163.com (S.S.); gongdf@imm.ac.cn (D.G.); wangranran@imm.ac.cn (R.W.); luy@imm.ac.cn (Y.L.); 2 Beijing Key Laboratory of Drug Targets Identification and Drug Screening, Institute of Materia Medica, Chinese Academy of Medical Sciences and Peking Union Medical College, Beijing 100050, China; liuruiqi@imm.ac.cn (R.L.); yeahchendi@163.com (D.C.); yuantianyi@imm.ac.cn (T.Y.); shoubaowang@imm.ac.cn (S.W.); 3 Beijing Key Laboratory of Polymorphic Drugs, Institute of Materia Medica, Chinese Academy of Medical Sciences and Peking Union Medical College, Beijing 100050, China; xingc@imm.ac.cn

**Keywords:** puerarin, pyroptosis, P2X7 receptor, H9C2 cell, RAW264.7 macrophages, diabetic cardiomyopathy

## Abstract

Diabetic cardiomyopathy (DCM) is a critical complication of long-term chronic diabetes mellitus, and it is characterized by myocardial fibrosis and myocardial hypertrophy. Previous studies have shown that the pyroptosis pathway was significantly activated in DCM and may be related to the P2X7 receptor. However, the role of the P2X7 receptor in the development of DCM with pyroptosis is still unclear. In this study, we aimed to explore the mechanism of puerarin and whether the P2X7 receptor can be used as a new target for puerarin in the treatment of DCM. We adopted systematic pharmacology and bioinformatic approaches to identify the potential targets of puerarin for treating DCM. Additionally, we employed D-glucose-induced H9C2 rat cardiomyocytes and lipopolysaccharide-treated RAW264.7 mouse mononuclear macrophages as the in vitro model on DCM research, which is close to the pathological conditions. The mRNA expression of cytokines in H9C2 cells and RAW264.7 macrophages was detected. The protein expressions of NLRP3, N-GSDMD, cleaved-caspase-1, and the P2X7 receptor were investigated with Western blot analysis. Furthermore, molecular docking of puerarin and the P2X7 receptor was conducted based on CDOCKER. A total of 348 puerarin targets and 4556 diabetic cardiomyopathy targets were detected, of which 218 were cross targets. We demonstrated that puerarin is effective in enhancing cardiomyocyte viability and improving mitochondrial function. In addition, puerarin is efficacious in blocking NLRP3-Caspase-1-GSDMD-mediated pyroptosis in H9C2 cells and RAW264.7 cells, alleviating cellular inflammation. On the other hand, similar experimental results were obtained by intervention with the P2X7 receptor antagonist A740003, suggesting that the protective effects of puerarin are related to the P2X7 receptor. The molecular docking results indicated key binding activity between the P2X7 receptor and puerarin. These findings indicate that puerarin effectively regulated the pyroptosis signaling pathway during DCM, and this regulation was associated with the P2X7 receptor.

## 1. Introduction

Diabetic cardiomyopathy (DCM) is a complication of diabetes without coronary heart disease or hypertension as an underlying cause. In DCM, the myocardial structure and function are impaired, including ventricular dysfunction, hypertrophy of cardiomyocytes, myocardial apoptosis, interstitial fibrosis, and dysregulation of metabolism. It is the main reason for the high incidence of heart failure and high mortality rates in diabetic patients [1]. The typical pathogenesis of DCM includes inflammation, oxidative stress, mitochondrial dysfunction, apoptosis, autophagy and myocardial fibrosis [2]. Recently, inflammation has been reported to play a critical role in diabetic cardiomyopathy, which may be a potential therapeutic target for diabetic cardiomyopathy [3]. Pyroptosis is a type of programmed cell death related to the activation of inflammation [4,5]. It is manifested by the continuous expansion of cells until the cell membrane ruptures, resulting in the release of contents and activation of a strong inflammatory response [6]. It has been reported that hyperglycemia activates nucleotide-binding oligomerization domain-like receptor pyrin domain-containing (NLRP)3 inflammasome (NLRP3), thus promoting pro-caspase-1 to caspase-1 [7]. The cleaved-caspase-1 is then capable of converting the pro-interleukin-1β (pro-IL-1β) and pro-interleukin-18 (pro-IL-18) into matured IL-1β/ IL-18. The cleaved N-terminal of gasdermin D (N-GSDMD) is essential in the process of pyroptosis because it can promote the secretion of matured IL-1β and damage the plasma membrane [8]. Relevant studies have shown that by regulating the release of inflammasomes and cytokines in the process of pyroptosis, as well as the expression of related genes, the occurrence and development of diabetic cardiomyopathy can be slowed down [9,10,11,12]. Further research on inflammation and pyroptosis can provide a new direction for the treatment of diabetic cardiomyopathy.

The death of cardiomyocytes is the basic change of cardiomyopathy, which can trigger myocardial remodeling and lead to left ventricular dysfunction, leading to DCM and heart failure [13]. Intracellular hyperglycemia is observed in the diabetic microvasculature [14]. Abnormal glucose metabolism caused by hyperglycemia is a key initiating factor of DCM [15]. With the progression of the disease, oxidative stress damage in myocardial tissue increases, excessive reactive oxygen species increase the expression levels of various inflammatory factors, and a long-term inflammatory environment will induce tissue and cell damage [16]. However, the exact mechanism of DCM is not fully elucidated. Recent studies have shown that pyrodeath may be closely related to the disease [17]. The H9C2 cell is a permanent cell line derived from the embryonic rat heart, which retains the characteristics of cardiomyocytes well.

Macrophage, known as one of the most dominant and widely distributed inflammatory cells, is involved in the initiation and maintenance of the acute inflammatory response [18]. Lipopolysaccharide (LPS), a pivotal inducer in inflammatory processes, is essentially important to pathogen-induced inflammation [19]. LPS promotes inflammatory pathogenesis by triggering the acute massive accumulation of neutrophils and the release of inflammatory mediators, such as IL-1β and IL-18 [20]. In addition, an appropriate amount of NO (nitric oxide) is beneficial for regulating considerable physiological conditions, whereas the sustained over-expression of NO is believed to be dangerous to humans [21]. Therefore, the regulation of the NO amount is a promising therapeutic strategy for many inflammatory diseases. Cells treated with LPS are considered a canonical model of inflammation research.

The P2X7 receptor (P2X7R) is an ATP-gated non-selective cation channel localized to mitochondria. Its main physiological function is to form ion channels and plasma membrane pores on the cell membrane surface, thereby participating in cell signal transduction, cytokine secretion, and mediating cell proliferation [22,23]. P2X7 receptor contributes to chronic inflammation in different disease models, including pain, neurodegeneration, acute pancreatitis, and atherosclerosis [24,25]. In this prospect, P2X7 receptor inhibitors have received great attention as a new therapeutic target. Studies have shown that P2X7 receptors play an important role in cardiovascular disease, especially ischemic injury. A large amount of extracellular ATP activates the P2X7 receptor, causes K^+^ efflux, regulates the assembly of NLRP3 inflammasomes, activates caspase-1, and secretes pro-inflammatory factors, thereby aggravating the phenotype of heart disease [26].

Puerarin, also known as dihydroxyisoflavone, isolated from the root of traditional Chinese medicine pueraria lobata, possesses a series of beneficial activities on cardiovascular diseases, such as myocardial infarction [27]. It was reported that puerarin prevents diabetic cardiomyopathy in vivo and in vitro by inhibition of inflammation [28].

Network pharmacology is a method to predict the composition of active ingredients and disease targets of compounds at the system level, and to establish a multi-level network such as drug–target–disease. Molecular docking utilizes flexible and semi-flexible docking to assess receptor–ligand interaction forces, thereby predicting receptor–ligand binding patterns and affinities [29].

Some studies have shown that the P2X7 receptor mediates NLRP3 inflammasome activation, thereby inducing the release of IL-1β and other cytokines, and regulating pyroptosis [30,31]. Previous animal experiments [32] showed that treatment with puerarin significantly restored cardiac function in diabetic cardiomyopathy rats by improving mitochondrial respiration, suppressing myocardial inflammation, and maintaining the structural integrity of the cardiac muscle. It was found that the mechanism of action of puerarin may be through inhibiting the expression of the P2X7 receptor and reducing myocardial pyroptosis. However, no studies have shown that puerarin exerts its anti-diabetic cardiomyopathy effect through the P2X7 receptor. This experimental study uses network pharmacology molecular docking technology; in addition, LPS-induced RAW264.7 cells and D-glucose-induced H9C2 cells were used as research models to approximate the pathological conditions of DCM. We explored the mechanism of action of puerarin in the treatment of DCM and verified whether the protective effect of puerarin on DCM is related to the regulation of the P2X7 receptor, providing an experimental basis for the clinical treatment of DCM.

## 2. Results

### 2.1. GO and KEGG Enrichment Analysis of Core Genes for Puerarin against Diabetic Cardiomyopathy

**Figure 1:**
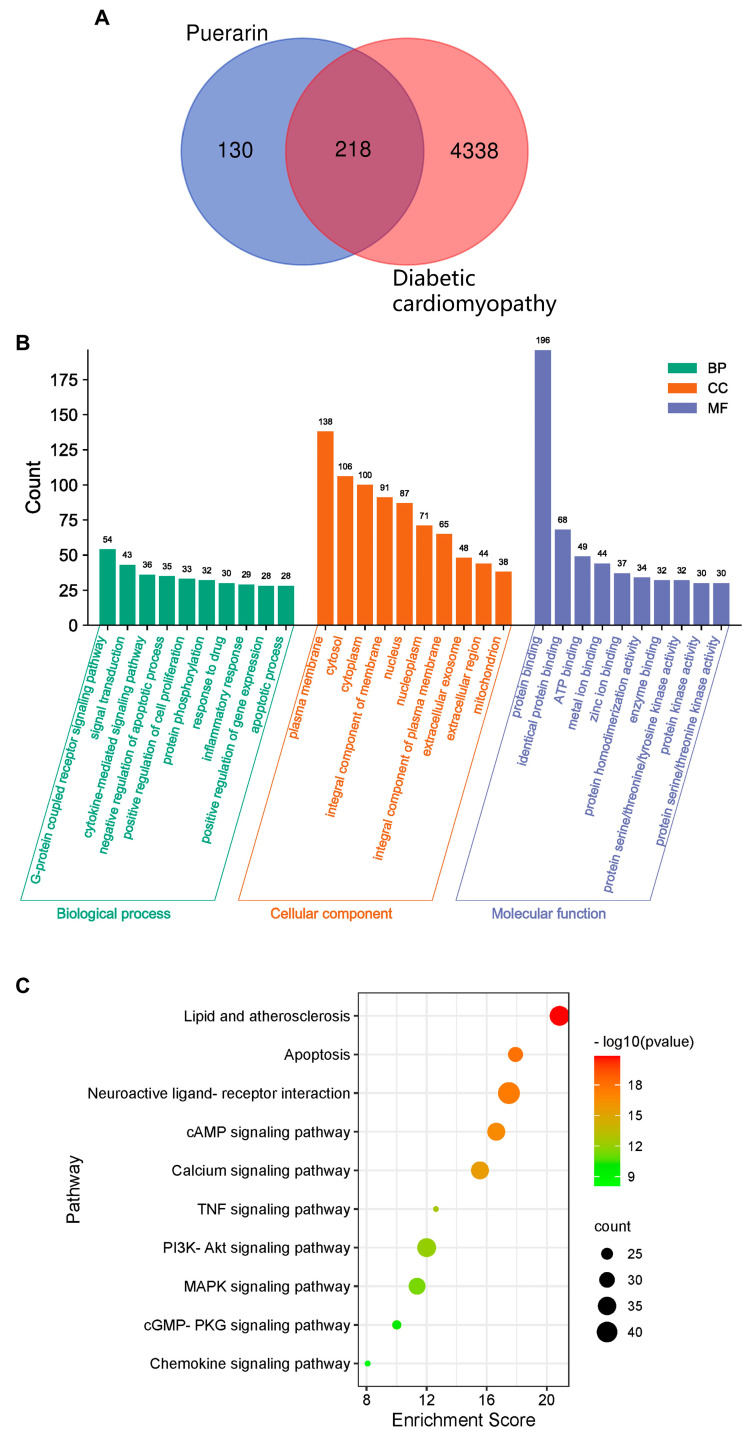
Network pharmacology. (**A**) Venn diagram of puerarin (blue) and diabetic cardiomyopathy genes (red), (**B**) GO analysis, and (**C**) top ten KEGG pathways. The color scales indicate the different thresholds for the *p*-values, and the sizes of the dots represent the number of genes corresponding to each term.

Based on the structure similarity algorithm and data mining, 348 pharmacological targets of puerarin were identified. Our results reveal that the DisGeNet, GenCLiP, and Comparative Toxicogenomics databases had a total of 4556 diabetic cardiomyopathy targets after removing duplicate targets. To clarify the relationship between puerarin and DCM, EXCEL was used to merge the targets found in puerarin with DCM, and finally, 218 intersection targets were obtained, as shown in Figure 1A, and then a Venn diagram was constructed to visualize the data. The 218 potential targets of puerarin to treat DCM were uploaded to the David database for enrichment analyses of biological processes, molecular functions, cellular components, and KEGG pathways (Figure 1B). The results showed that the cellular component was mainly enriched in the plasma membrane, cytosol, cytoplasm, and integral components of the membrane. Their main molecular functions were associated with protein binding, ATP binding, and metal ion binding. The KEGG results showed that the targets were mainly enriched in the following: the neuroactive ligand–receptor interaction, calcium signaling pathway, and chemokine signaling pathway (Figure 1C). According to the *p*-value, the top ten GO terms and top ten KEGG pathways were plotted in an online platform for data visualization.

### 2.2. Effects of Puerarin on the Viability of H9C2 Cells

**Figure 2:**
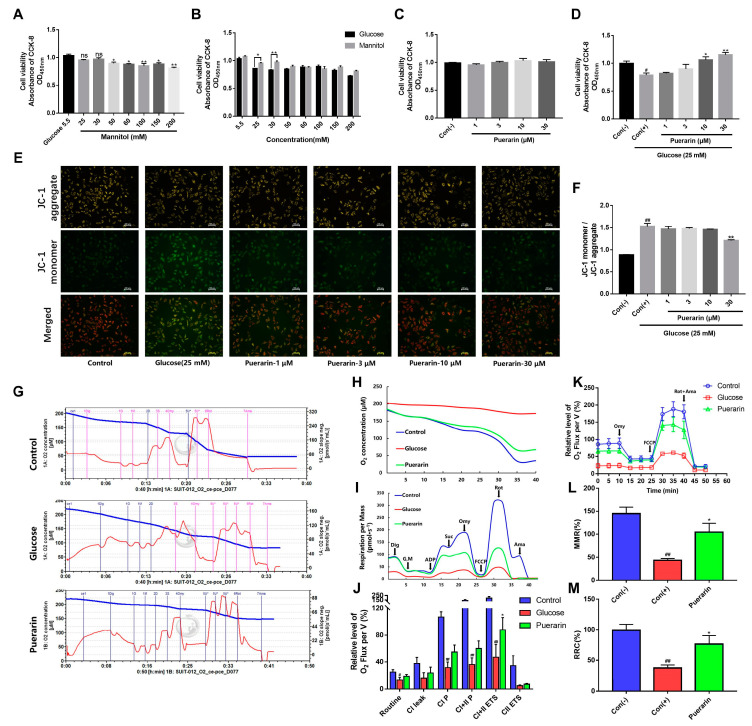
Puerarin reverses D-glucose-induced cytotoxicity and mitochondrial dysfunction in H9C2 cells. (**A**) Effects of mannitol on cell viability in H9C2 cells for 24 h. (**B**) The difference between mannitol and glucose on cell viability in H9C2 cells for 24 h. (**C**) Effects of puerarin on H9C2 cells viability under normoxia conditions. (**D**) Effects of puerarin on cell viability in D-glucose-induced H9C2 cell injury. (**E**) Fluorescence image of JC-1 staining in H9C2 cells exposed to 25 mmol/L D-glucose for 24 h. Scale bar: 100 μm. (**F**) Quantitative analysis of the ratio of red fluorescence to green fluorescence in (**E**). (**G**) Effects of puerarin (30 μmol/L) on the mitochondrial complex of H9C2 cells induced by D-glucose (25 mmol/L) for 24 h. (**H**,**I**) The representative profile of O_2_ concentration change and relative level of O_2_ flux per volume of H9C2 cells in the high glucose model. (**J**) The summarized data of mitochondrial respiration in glucose and puerarin group, including routine, CI, and CI plus CII oxidative phosphorylation, CI and CII leak, as well as CI plus CII electron transfer system. (**K**) Representative profiles and statistical data of mitochondrial respiration of H9C2 cells treated with glucose (25 mmol/L, 24 h). (**L**,**M**) Effects of puerarin on routine respiration, maximum mitochondrial respiration (MMR), and residual respiration consumption (RRC) of H9C2 cells induced by D-glucose (25 mmol/L, 24 h). Data are shown as mean ± SEM (n = 3). ^#^ *p* < 0.05, ^##^ *p* < 0.01 vs. control, * *p* < 0.05, ** *p* < 0.01 vs. model.

We measured D-glucose-induced cytotoxicity in H9C2 cells with different concentrations of D-glucose. At the same time, we used the same concentration of mannitol as a control to eliminate the effect of increased osmotic pressure. As shown in Figure 2A, mannitol at 25 and 30 mmol/L had no significant effect on the cells. According to the results in Figure 2B, D-glucose at 25 and 30 mmol/L significantly increased cytotoxicity compared to the control group. Therefore, we used 25 mmol/L D-glucose in this study, because the cell damage at this concentration is caused by glucose, not by osmotic pressure. H9C2 cells were incubated with puerarin for 24 h, and puerarin concentration (1, 3, 10, 30 μmol/L) had no significant effect on H9C2 cells (Figure 2C). As shown by the results in Figure 2D, puerarin (1–30 μM) pre-incubation for 2 h can protect cardiomyocytes against hyperglycemic damage in a concentration-dependent manner, for which the effects of 10 and 30 μM puerarin are most significant.

### 2.3. Effects of Puerarin on the Mitochondrial Membrane Potential of H9C2 Cells

We determine the mitochondrial membrane potential by the confocal laser scanning microscope and JC-1 probe staining method. As shown in Figure 2E, following 24 h exposure to D-glucose 25 mmol/L, there was a significant increase in the green fluorescence intensity of the JC-1 probe, and the red fluorescence intensity was significantly weakened (*p* < 0.01), indicating that the mitochondrial membrane potential decreased. Varying concentrations of puerarin (1, 3, 10, and 30 μmol/L) decreased the green fluorescence intensity and increased the red fluorescence intensity of the JC-1 probe in a concentration-dependent manner with the greatest effect at the concentration of 30 μmol/L (Figure 2F).

### 2.4. D-Glucose Stimulus Inhibits the Mitochondrial Respiratory Function of H9C2 Cells

H9C2 cells were pretreated with a concentration of puerarin (30 μmol/L) for 2 h followed by D-glucose at 25 mmol/L for 24 h. We determined the mitochondrial respiratory function related to complex I and II in H9C2 cells using Oxygraph-2k high-resolution respirometry. The oxygen consumption was measured in intact cells with a phosphorylation control protocol. Oligomycin (Omy, 1 μmol/L), FCCP (1, 2, 3, 4 μmol/L), and rotenone (Rot, 1 μmol/L) combined with antimycin A (Ama 1 μmol/L) were added sequentially to H9C2 cells.

Compared with the control group, the model group showed an overall reduction in mitochondrial function (Figure 2G–I); furthermore, routine, CI P, CI + II P, and CI + IIETS parameters were markedly reduced (Figure 2J). These results indicated that the function of mitochondrial respiratory complex I/II in H9C2 cells was impaired in diabetic cardiomyopathy. The pre-incubation of puerarin can significantly improve cellular respiratory function, and the CI + CII electron transfer system (CI + II ETS) is the most significant (Figure 2J).

We treated H9C2 cells cultured with D-glucose (25 mmol/L) for 24 h and measured the mitochondrial function. We found that H9C2 cells displayed a general reduction in respiration under D-glucose stimulation. Specifically, as shown in Figure 2K–M, all the cellular mitochondrial functional parameters, including routine respiration, maximum mitochondrial respiration (MMR), and residual respiration consumption (RRC), were markedly impaired in cardiomyocytes stimulated by D-glucose (25 mmol/L). However, the pre-incubation of puerarin (30 μmol/L) can gently protect cells and improve mitochondrial function.

### 2.5. Puerarin Inhibits the Inflammatory mRNA Expression of H9C2 Cardiomyocytes

**Figure 3:**
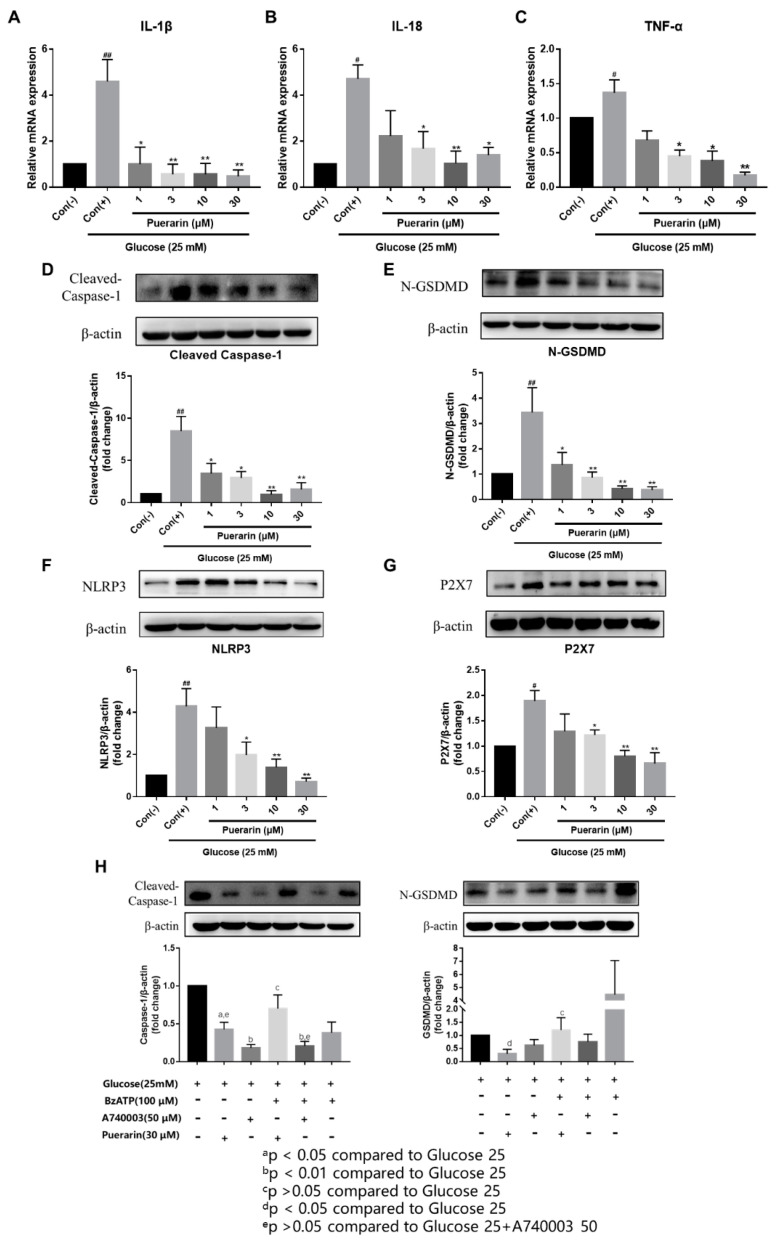
Puerarin inhibited pyroptosis in H9C2 cells induced with D-glucose. Effects of puerarin on IL-1β (**A**), IL-18 (**B**), and TNF-α (**C**) mRNA expression of H9C2 induced with glucose (25 mM) for 24 h. Effects of puerarin (1–30 μmol/L) on the expression of (**D**) cleaved-caspase-1, (**E**) N-GSDMD, (**F**) NLRP3, and (**G**) the P2X7 receptor. (**H**) Puerarin decreases pyroptosis-related protein expression from H9C2 cells. Cells were pretreated with BzATP (100 μmol/L) and/or A740003 (50 μmol/L) for 2 h prior to puerarin, and with or without puerarin (30 μmol/L) for 24 h. LPS ± BzATP induced Caspase-1 and GSDMD expression that was inhibited by puerarin, similar to the effect of A740003. Relative density analysis of the protein bands was shown by the Western blot with β-actin as a control. Data are shown as mean ± SEM (n = 3). ^#^ *p* < 0.05, ^##^ *p* < 0.01 vs. control, * *p* < 0.05, ** *p* < 0.01 vs. model.

We subsequently measured IL-1β, IL-18, and TNF-α mRNA expression in H9C2 cells 24 h after D-glucose stimulation. We detected a significant increase in IL-1β (Figure 3A), IL-18 (Figure 3B), and TNF-α (Figure 3C) mRNA expression from H9C2 cells, compared to the control media. In comparison, puerarin inhibits inflammatory mRNA expression in a concentration-dependent manner (1–30 μmol/L).

### 2.6. Effects of Puerarin on NLRP3-Caspase-1-GSDMD Mediated Pyroptosis in H9C2 Cells

Pyroptosis plays an essential role in myocardial cell damage during myocardial infarction. Previous studies have shown that the NLRP3-Caspase-1-GSDMD pathway is involved in high-glucose-induced injury. Therefore, we evaluated whether puerarin protects H9C2 cells exposed to D-glucose. We identified that D-glucose significantly increased cleaved-caspase-1 (Figure 3D), N-GSDMD (Figure 3E), NLRP3 (Figure 3F), and P2X7 receptor (Figure 3G) expression, and puerarin significantly decreased NLRP3, N-GSDMD, cleaved-caspase-1, and P2X7 receptor expression in a concentration-dependent manner.

### 2.7. Effects of Puerarin on the Viability of RAW264.7 Cells

**Figure 4:**
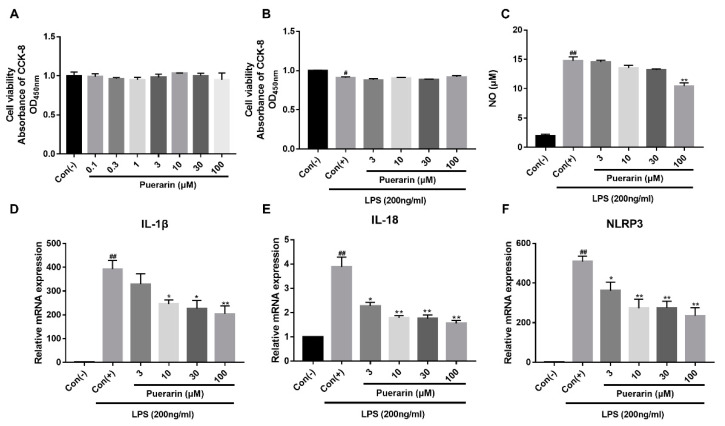
Puerarin attenuates LPS-induced inflammation in RAW264.7 cells. (**A**) Effects of puerarin on RAW264.7 cell viability under normoxia conditions. (**B**) Effects of puerarin on cell viability in RAW264.7 cells for 48 h. (**C**) Effects of puerarin on the NO production of RAW264.7 cells induced by LPS. Puerarin downregulates IL-1β (**D**), IL-18 (**E**), and NLRP3 (**F**) mRNA expression. Data are shown as mean ± SEM (n = 3). ^#^ *p* < 0.05, ^##^ *p* < 0.01 vs. control, * *p* < 0.05, ** *p* < 0.01 vs. model.

RAW264.7 cells were treated with puerarin (3, 10, 30, and 100 μmol/L, Figure 4A) for 48 h, and no significant difference was shown among the groups regarding viability. Puerarin can significantly decrease NO production at 100 μmol/L (Figure 4B). The results also indicated that the reduction in NO by puerarin was not achieved by regulating cell viability (Figure 4C).

### 2.8. Puerarin Inhibits the Inflammatory mRNA Expression of RAW264.7 Macrophages

We also found that there is a significant increase in IL-1β (Figure 4D), IL-18 (Figure 4E), and NLRP3 (Figure 4F) mRNA expression in LPS-stimulated RAW264.7 cells. Puerarin also inhibits the inflammatory mRNA expression of RAW264.7 cells in a concentration-dependent manner (3–100 μmol/L).

### 2.9. Effects of Puerarin on Key Proteins That Mediate Pyroptosis Pathways in Treated RAW264.7 Macrophages

**Figure 5:**
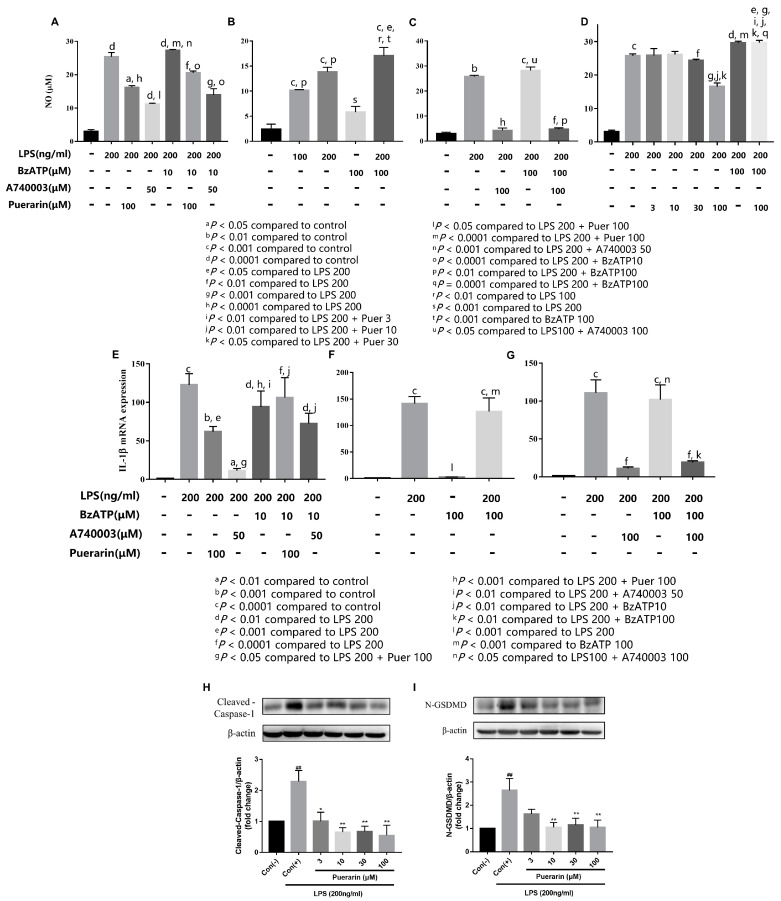
Puerarin decreased NO (**A**–**D**) production and mRNA expression (**E**–**G**) in RAW264.7 cells stimulated with LPS (100, 200 ng/mL), BzATP (10 μmol/L or 100 μmol/L), A740003 (50 μmol/L or 100 μmol/L), and with or without puerarin (3, 10, 30, 100 μmol/L) for 48 h. LPS ± BzATP induced NO production that was inhibited by puerarin, similar to the effect of A740003. Effects of puerarin (3–100 μmol/L) on the expression of (**H**) cleaved-caspase-1 and (**I**) N-GSDMD. Data are shown as mean ± SEM (n = 3). ^##^ *p* < 0.01 vs. control, * *p* < 0.05, ** *p* < 0.01 vs. model.

In the present study, we also examined the expression of proteins involved in pathways of pyroptosis. As shown in Figure 5H,I, LPS resulted in significantly high levels of cleaved-caspase-1 and N-GSDMD protein. While puerarin inhibited the expression of pyroptosis protein, puerarin at higher concentrations had a more significant inhibitory effect.

### 2.10. P2X7 Receptor Plays an Important Role in Puerarin Action against D-Glucose-Induced H9C2 Cell Injury and LPS-Induced Inflammation of RAW 264.7 Macrophages

Based on the above studies, we hypothesized that the P2X7 receptor is significantly activated, leading to NLRP3/Caspase-1-mediated pyroptosis. In order to prove the hypothesis, we adopted A740003, a specific P2X7 receptor antagonist, and BzATP, a P2X7 receptor agonist that induces inflammatory–excitotoxicity with LPS [33], to further investigate the effect of the P2X7 receptor in H9C2 cells with D-glucose and RAW264.7 cells with LPS. We subsequently measured NO production and IL-1β mRNA expression following 48 h of LPS exposure (200 ng/mL) in RAW 264.7 cells. In addition, we examined whether puerarin inhibited NO production and IL-1β mRNA expression, and whether puerarin acted through the P2X7 receptor.

Compared with the control group, the production of NO in macrophages was significantly increased after LPS stimulation. As the results showed in Figure 5A–D, after puerarin intervention, the production of NO decreased significantly. Experiments demonstrated that 50 μmol/L A740003 significantly inhibited NO production under inflammation–excitotoxic conditions after exposure to LPS (200 ng/mL) and BzATP (10 μmol/L) for 48 h. Similarly, 100 μmol/L puerarin significantly decreased NO production under inflammatory–excitotoxic conditions following 48 h exposure to LPS (200 ng/mL) and BzATP (10 μmol/L). We did not detect a statistically significant difference between LPS (200 ng/mL) and LPS (200 ng/mL) + BzATP (10 μmol/L); thus, we chose BzATP (100 μmol/L) to generate the excitotoxic stimuli in our further experiments. As little as 100 μmol/L of BzATP alone can cause little NO production. In addition, 100 μmol/L A740003 significantly inhibited NO production under inflammatory–excitotoxic conditions following 48 h exposure to LPS (200 ng/mL) and BzATP (100 μmol/L). After 48 h of LPS (100 ng/mL) and BzATP (100 μmol/L), co-incubation of 100 μmol/L puerarin significantly reduced inflammation and NO production under inflammation–excitotoxic conditions.

We focused our experiments on the detection of IL-1β (Figure 5E–G). The result is consistent with Figure 5A–D. The P2X7 receptor is an essential upstream target of pyroptosis. As the results showed, puerarin can reduce the expression of pyroptosis-related proteins in H9C2 cells in a concentration-dependent manner (Figure 3D–G). We focused our study on the interested N-GSDMD and cleaved-caspase-1, and observed whether puerarin regulates N-GSDMD and cleaved-caspase-1 in H9C2 cells through the P2X7 receptor. We chose the P2X7 receptor agonist BzATP and the antagonist A740003. According to the results in Figure 3H, puerarin (D-glucose + puerarin) can significantly inhibit the expression of cleaved-caspase-1 and N-GSDMD expression, which is consistent with the inhibitor A740003. The agonist BzATP (D-glucose + BzATP) can up-regulate cleaved-caspase-1 expression, significantly increasing the expression of N-GSDMD. We found that puerarin (D-glucose + puerarin + BzATP) loses its ability to down-regulate the expression of cleaved-caspase-1 and N-GSDMD after adding the agonist BzATP. These results further illustrated that puerarin inhibits pyroptosis via downregulation of the P2X7 receptor on H9C2 cells. While the inhibitor A740003 (D-glucose + A740003 + BzATP) can still significantly inhibit the expression of cleaved-caspase-1 and N-GSDMD, indicating that the binding ability of A740003 to P2X7 receptor is stronger than that of BzATP. In conclusion, puerarin down-regulates the pyroptosis-mediated protein of H9C2 cells, inhibits IL-1β mRNA expression, and reduces NO production in RAW264.7 cells through the P2X7 receptor.

### 2.11. Molecular Docking of Puerarin and P2X7 Receptor

We downloaded the mol2 format file (MOL012297) of the structure of puerarin from the TCMSP platform, and the P2X7 receptor (PDB ID: 5U1Y) format file from the PDB (protein data bank). We then imported them into the DS BIOVIA Discovery Studio Client 2016 v16. 1 software for CDOCKER molecular docking (Table 1). The P2X7 receptor (PDB ID: 5U1Y) interacted with four ligands to form four active sites, which were molecularly docked to puerarin and compared with its original ligand binding energies.

**Figure 6:**
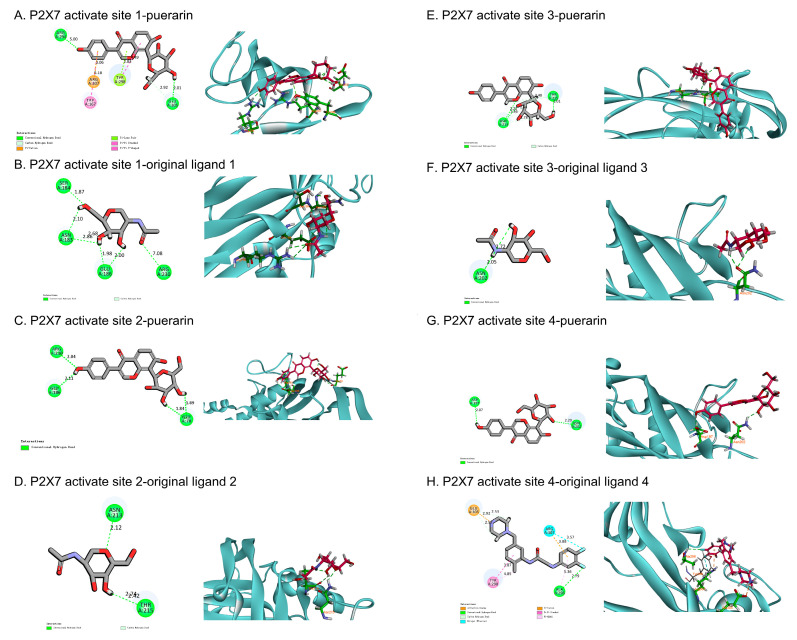
Molecular docking diagram. Molecular models of the binding of puerarin with the P2X7 receptor and the results are shown as 3D diagrams. (**A**) P2X7 receptor activate site 1–puerarin, (**B**) P2X7 receptor activate site 1–original ligand 1, (**C**) P2X7 receptor activate site 2 –puerarin, (**D**) P2X7 receptor activate site 2–original ligand 2, (**E**) P2X7 receptor activate site 3–puerarin, (**F**) P2X7 receptor activate site 3–original ligand 3, (**G**) P2X7 receptor activate site 4–puerarin, and (**H**) P2X7 receptor activate site 4–original ligand 4.

As shown in Figure 6, the -CIE of puerarin and the P2X7 receptor in the first three active sites is higher than that of the original ligand, indicating that its binding ability to the P2X7 receptor is better than that of the original ligand. Among them, in the first active site, the -CIE of puerarin and P2X7 receptor is 29.205 kcal/mole, the interaction binding energy is low, the binding is relatively tight, and multiple hydrogen bonds and pi–pi interactions are formed. At the fourth active site, the -CIE of the original ligand and P2X7 receptor is 33.258 kcal/mole, the interaction binding energy is the lowest, and the binding is the tightest, forming multiple attractive charges, hydrogen bonds, and pi–alkyl hydrophobic interactions (Table 1). This indicates that puerarin binds to the P2X7 receptor with abundant active sites, high binding ability, and strong interaction.

## 3. Discussion

In this study, based on network pharmacology, we found that puerarin principally regulates diabetic cardiomyopathy through the neuroactive ligand–receptor interaction, calcium signaling pathway, and chemokine signaling pathway, which reflects the side that diabetic cardiomyopathy and puerarin are closely related to inflammatory death—pyroptosis. Next, we searched for the upstream targets of pyroptosis and found that the P2X7 receptor was closely related to the cellular components and pathways discovered by network pharmacology. In previous animal studies, puerarin has been confirmed to affect P2X7 receptor expression in the myocardial tissue of diabetic cardiomyopathy rats. Therefore, we boldly predicted that the P2X7 receptor might become a novel target for puerarin in the treatment of diabetic cardiomyopathy. Next, we verified that puerarin regulates pyroptosis through the P2X7 receptor to improve diabetic cardiomyopathy through the vitro model of DCM and molecular docking.

Both clinical and basic studies have confirmed that high glucose can directly cause cardiomyocyte dysfunction, leading to systolic and diastolic dysfunction in diabetic hearts [34]. However, the molecular mechanism of cardiomyocyte dysfunction induced by high glucose is still unclear. Our data showed that puerarin is non-toxic to H9C2 cells at concentrations of no more than 30 µmol/L (Figure 2C), and the protection conferred by puerarin on H9C2 cells that are exposed to D-glucose is concentration-dependent (Figure 2D). We also identified that puerarin could increase the mitochondrial membrane potential of H9C2 cells in glucose damage, which indicated that puerarin alleviates glucose-induced early apoptosis (Figure 2E,F). These results confirmed the potent protective effects of puerarin in the H9C2 cells exposed to D-glucose.

Many studies have reported that mitochondria play an important role in the survival of cardiomyocytes, and the imbalance of mitochondrial function can directly lead to pyroptosis. It has been reported that complex I/II activity was mainly diminished in severe heart diseases in vivo. Schipper et al. found that ischemic patients have an aberrant mitochondrial function, highlighted by a lowered respiratory control rate, and all ratios involving complex I were affected [35]. Galan et al. reported that cardiac myocytes, after myocardial ischemia, had reduced mitochondrial respiration and glycogen accumulation, as well as complex I and II activity [36]. In addition, the P2X7 receptor is localized to mitochondria and is a key regulator of mitochondrial energy metabolism [37]. Therefore, we focused on the detection of puerarin on mitochondrial respiratory function in cardiomyocytes. It was observed that the mitochondrial respiration capacity, MMR and RRC are significantly reduced in H9C2 cells stimulated by D-glucose (25 mmol/L). However, pretreatment with puerarin (30 µmol/L) can improve mitochondrial function and significantly increase the MMR and RRC (Figure 2G–M), suggesting that the protective effect of puerarin on H9C2 cells may be related to the regulation of mitochondrial function.

Cardiac inflammation is an early and actively involved process in the development of heart failure during DCM. To explore the potential anti-inflammatory effects of puerarin, the LPS-induced generations of these cytokines with the treatment of puerarin were examined. We characterized that puerarin-induced attenuation of inflammatory injury is concentration-dependent, such that a higher concentration (100 μmol/L) of puerarin is significantly superior in inhibiting NO production and inflammatory cytokine mRNA expression from RAW264.7 cells during the inflammation-induced cellular damage.

We identified that puerarin can significantly decrease the mRNA expression of IL-1β and IL-18 in both H9C2 and RAW264.7 cells (Figure 3A–C and Figure 4D–F). The data showed that the expression of NLRP3, cleaved-caspase-1, and N-GSDMD was effectively increased in H9C2 cells administrated with D-glucose when compared with the control group, while puerarin effectively down-regulates N-GSDMD and cleaved-caspase-1, which was due to the down-regulation of NLRP3 protein expression (Figure 3D–F). Further, the expression of the P2X7 receptor was also significantly increased in H9C2 cells administrated with D-glucose (Figure 3G). Thus, we deem that the activation of NLRP3/caspase-1-mediated pyroptosis is synchronous with the activation of the P2X7 receptor. Accordingly, we hypothesized that P2X7 receptor expression is strongly activated in H9C2 cells with hyperglycemia injury, causing NLRP3/caspase-1-mediated pyroptosis. In order to prove the hypothesis, we adopted A740003, a specific P2X7 receptor antagonist, or BzATP, a P2X7 receptor agonist [33], to further investigate the effect of the P2X7 receptor in H9C2 cells with D-glucose and RAW264.7 cells with LPS. Our study establishes the importance of puerarin in the overall inflammatory process involving RAW264.7 macrophages. Using 100 μmol/L A740003, we showed complete abrogation of LPS-induced inflammatory responses, specifically expression of IL-1β and production of NO. We also determined that this concentration of A740003 was sufficient to inhibit RAW264.7 inflammatory responses even in the presence of other P2X7 receptor agonists, such as BzATP. When we repeated our experiments with puerarin, we demonstrated decreased NO production and IL-1β expression when RAW264.7 cells were cultured with BzATP (10 and 100 μmol/L). Our data shows similar reductions in RAW264.7 cell NO production and IL-1β expression in culture conditions supplemented with puerarin in comparison to A740003. This supports our hypothesis that puerarin acts through the P2X7 receptor to inhibit RAW264.7 inflammatory responses.

We also demonstrated that the protection effects of puerarin in H9C2 cells were also through the P2X7 receptor, following the above treatment with BzATP and A740003. The expression of key proteins that mediate pyroptosis in treated H9C2 cells is shown in Figure 3H. As the results show, A740003 can effectively inhibit the expression of cleaved-caspase-1 when cultured with or without BzATP, and can be observed to inhibit the expression of N-GSDMD, but it is not significant. Our data also show that puerarin can significantly inhibit the expression of cleaved-caspase-1 and N-GSDMD expression in H9C2 cells treated with BzATP. Additionally, there is similar down-regulation in cleaved-caspase-1 and N-GSDMD expression in culture conditions supplemented with puerarin in comparison to A740003. This supports our hypothesis that puerarin acts through the P2X7 receptor to improve H9C2 cells. In addition, the molecular docking results indicate that puerarin binds to the P2X7 receptor with abundant active sites, high binding ability, and strong interaction. These results suggested that the P2X7 receptor can mediate the NLRP3/caspase-1-induced pyroptosis to regulate hyperglycemia changes and further supported that the P2X7 receptor can be a potential target for the treatment of diabetic cardiomyopathy.

## 4. Materials and Methods

### 4.1. Puerarin Target Collection

Targets of puerarin were collected from different databases. Based on structural similarity and data mining algorithms, targets of puerarin were exacted from the TCMSP, ChEMBL database (http://www.ebi.ac.uk/chembl/, accessed on 7 March 2022), Swiss Target Prediction (http://www.swiss targetprediction.ch, accessed on 7 March 2022), Drug Bank (http://www.drugbank.ca, accessed on 7 March 2022), and BindingDB (http://www.bindingdb.org/bind/index.jsp, accessed on 7 March 2022). Acquired targets were then mapped to the UniProt database (http://www.uniprot.org/, accessed on 7 March 2022) for normalization.

### 4.2. Identification of DCM-Related Targets

The DisGeNET platform (http://www.disgenet.org, accessed on 8 March 2022), GenCLiP 2.0 online tool (http://ci.smu.edu.cn/, accessed on 8 March 2022), and the Comparative Toxicogenomics Database (http://ctdbase.org/, accessed on 8 March 2022) were accessed to collect DCM-related genes using the search terms “diabetic cardiomyopathy” and reproducible targets were removed.

### 4.3. Gene Ontology (GO) and the Kyoto Encyclopedia of Genes and Genomes (KEGG) Enrichment Analysis of the Core Targets for Puerarin against DCM

Functional annotation was performed using GO enrichment and KEGG pathway analyses. The David online tool was used to thoroughly analyze enrichment information. Potential therapeutic targets of puerarin against DCM were supplied to the David tool to produce enriched GO terms and KEGG pathways. GO/KEGG terms with *p* < 0.05 were considered significantly enriched.

### 4.4. Cell Culture and Treatment

H9C2 cells were obtained from Sciencell Research Laboratories. Cells were cultured in Dulbecco’s Modified Eagle Medium (DMEM; Gibco, New York, OH, USA) supplemented with 10% of fetal bovine serum (FBS; Superfine, Hyclone, Logan, UT, USA), 100 U/mL penicillin, and 100 μg/mL streptomycin (Solarbio, Beijing, China) in humidified air at 37 °C with 5% CO_2_. All experiments were performed with cells kept in culture between three and six passages. When H9C2 cell populations reached 40–50% confluence, the cultures were exposed to D-glucose (Sigma–Aldrich, St. Louis, MO, USA) at a final concentration of 25, 30, 50, 60, 100, 150, 200 mmol/L (high glucose) and mannitol (Sigma–Aldrich, St. Louis, MO, USA) at the same final concentration as D-glucose (high osmotic pressure) for 24 h. The concentration of D-glucose was finally determined to be 25 mmol/L. In addition, some cultured cells were exposed to 5.5 mmol/L D-glucose as a control. Cells were pretreated with different levels of puerarin (1, 3, 10, and 30 μmol/L) for 2 h, followed by D-glucose (25 mmol/L) stimulation for another 24 h.

Murine macrophage cell line RAW264.7 was obtained from the American Type Culture Collection (ATCC, Rockville, MD, USA). The cells were cultured in Dulbecco’s modified Eagle’s medium (DMEM; Gibco, St. Katharinen, Germany) supplemented with 10% heat-inactivated fetal bovine serum (FBS, Superfine; Gibco, New York, OH, USA), 100 U/mL penicillin and 100 μg/mL streptomycin (Solarbio, Beijing, China) in a humidified air at 37 °C with 5% CO_2_. All experiments were performed with cells kept in culture between three and six passages. Briefly, RAW264.7 macrophages were exposed to lipopolysaccharides (LPS, Sigma–Aldrich, St. Louis, MO, USA) (200 ng/mL) with or without puerarin treatment (3, 10, 30, and 100 μmol/L) for 48 h. The morphology change was observed by a microscope (Olympus IX71, Tokyo, Japan).

### 4.5. Cell Viability Assay

The toxicity of puerarin and/or D-glucose on H9C2 and RAW264.7 cells with or without LPS was determined by the Cell Counting Kit-8 (CCK-8, Dojindo, Tokyo, Japan) assay according to the manufacturer’s instructions. In brief, following the above cell treatment protocol, cells were seeded into 96-well plates and cultured in DMEM containing 10% FBS for 24 h, and the medium was then replaced with serum-free medium for 12 h. Serum-starved cells were treated with different concentrations of puerarin followed by D-glucose or LPS. CCK-8 (diluted 1:10) solution was added to each well, followed by incubation with 5% CO_2_ at 37 °C for 1 h. The absorbance values were measured at 450 nm using a Molecular Devices SpectraMax M5 microplate reader (Shanghai, China). The assay was performed in triplicate at each concentration. Cell viability was expressed as a percentage of the untreated control.

### 4.6. Determination of Mitochondrial Membrane Potential

The cells were treated in the same way as mentioned above. After 24 h incubation under high-glucose conditions, the mitochondrial membrane potential was measured by a JC-1 fluorescent probe. Firstly, the supernatant was replaced with the JC-1 staining working solution (1×). After incubation in darkness for a further 20 min at 37 °C, cells were washed twice with JC-1 washing buffer. Finally, JC-1 monomer (excitation wavelength and emission wavelength of 490 and 530 nm) and JC-1 polymer (excitation wavelength and emission wavelength of 525 and 590 nm) were analyzed by the Molecular Devices SpectraMax M5 microplate reader. Images were subsequently taken and analyzed using a fluorescent inverted microscope (Tokyo, Japan).

### 4.7. Mitochondrial Respiratory Function Detection

Firstly, the O_2_ concentration in the chamber was calibrated according to the protocol. H9C2 cells were harvested via trypsin digestion and centrifuged at 800 rpm for 3 min at room temperature. Then, the pellet was resuspended in MiR05 (Sigma–Aldrich, St. Louis, MO, USA) for high-resolution respirometry. The MiR05 consisted of EGTA 0.5 mmol/L, lactobionic acid 60 mmol/L, MgCl_2_·6H_2_O 3 mmol/L, KH_2_PO_4_ 10 mmol/L, taurine 20 mmol/L, HEPES 20 mmol/L, D-sucrose 110 mmol/L, and bovine serum albumin (BSA) (fatty acid free) 5 g/L. Mitochondrial respiratory function was measured in a two-chamber titration injection respirometer (Oxygraph-2k; Oroboros Instruments, Innsbruck, Austria). The final density of the cell suspension in the chamber was approximately 1 × 10^6^ cells/mL. Data were recorded through DatLab software 5.2 (Oroboros Instruments, Innsbruck, Austria).

In order to detect the mitochondrial respiratory function of H9C2 cells, two classical protocols were adopted. In intact cells, we used the protocol with substrate–uncoupler–inhibitor titrations. In detail, the intact cell measure protocols were as follows: routine respiration (Routine) was followed by manual titration of oligomycin (Omy, Sigma–Aldrich, St. Louis, MO, USA, 2 μg/mL) to induce the non-phosphorylating leak state, then titrate uncoupler (Sigma–Aldrich, St. Louis, MO, USA, 1 mM FCCP) was manually titrated until the maximum noncoupled flux (capacity of the electron transfer system, ETS) was induced. Finally, the maximal mitochondrial respiration (MMR) and reserve respiratory capacity (RRC) were calculated.

In permeabilized cells, we used the protocol with substrate–uncoupler–inhibitor titrations. After respiration was stabilized for a short time, routine respiration was measured. Then, digitonin (Dig, Sigma–Aldrich, St. Louis, MO, USA, 150 µg/10^6^ cells) was applied for plasma membrane permeabilized. Glutamate (G, Sigma–Aldrich, St. Louis, MO, USA, 5 mmol/L) and malate (M, Sigma–Aldrich, St. Louis, MO, USA, 2 mmol/L) were used to induce the respiratory leak state of complex I (CI Leak). Then, 5 mmol/L ADP (Sigma–Aldrich, St. Louis, MO, USA) was added to detect the oxidative phosphorylation (OXPHOS) capacity of complex I (CI P). Maximal OXPHOS capacity was induced by succinate (Sigma–Aldrich, St. Louis, MO, USA, 100 mmol/L), including both CI and complex II OXPHOS capacity (CII, CI + IIP). Next, oligomycin and FCCP (Sigma–Aldrich, St. Louis, MO, USA) titrations were used for the maximal uncoupled respiratory capacity of the electron transfer system (ETS) (CI + II ETS). CII-related uncoupled respiratory function (CII ETS) was detected after the addition of rotenone (Rot, 0.5 µmol/L). Finally, antimycin A (Ama, Sigma–Aldrich, St. Louis, MO, USA, 2.5 µmol/L) was given for residual oxygen consumption evaluation [38].

### 4.8. Determination of Nitric Oxide (NO) Production

1 × 10^4^ cells/well RAW264.7 cells were seeded in 96-well plates and incubated overnight, followed by the treatment with puerarin (3, 10, 30, and 100 μmol/L) and LPS (200 ng/mL) stimulation for 48 h. NO in culture medium was measured directly using a commercially available kit (Beyotime, Shanghai, China) based on the Griess reaction. Culture supernatants (50 μL) were mixed with 50 μL Griess I reagent for 10 min at room temperature, then mixed with 50 μL Griess II reagent for 10 min at room temperature. The absorbance values were measured at 540 nm on a Molecular Devices SpectraMax M5 microplate reader.

### 4.9. Extraction of RNA and Quantitative Real-Time Polymerase Chain Reaction (RT-qPCR)

Total RNA was extracted using trizol. Complementary DNA was reverse transcribed from mRNA using a MonScript™ 5× RTIII All-in-One Mix (MR05001, Monad Biotech Co., Ltd., Suzhou, China) by priming for 10 min at 25 °C, reverse transcription (RT) for 15 min at 55 °C, and RT inactivation for 5 min at 85 °C. A qPCR reaction was performed on a CFX96 Touch Real-Time PCR Detection System (Bio-Rad, Hercules, CA, USA), with SYBR Premix Ex Taq II (Tli RNaseH Plus, Vazyme, Nanjing, China) in 20 μL reactions for 50 cycles, using the manufacturer’s protocol for temperature cycling (Bio-Rad, Hercules, CA, USA) and denaturation at 95 °C for 10 secs and extension at 60 °C for 30 s. Primers for H9C2 and RAW264.7 cells are shown in Table 2. Primers are designed by PubMed.

The results were subjected to melting curve analysis, and the data were analyzed using the 2^−ΔΔCq^ method. Experiments were performed in triplicate.

### 4.10. Western Blot Analysis

The H9C2 cells pretreated with puerarin (1, 3, 10, and 30 μmol/L), with or without high glucose and with BzATP or/and A740003 were collected, washed twice with cold PBS and lysed in a RIPA buffer (Beyotime, Shanghai, China). The total protein concentration was measured using a bicinchoninic acid (BCA) protein assay kit (CWBIO, Beijing, China). Equivalent amounts of 50 μg protein samples were loaded into SDS polyacrylamide gels and transferred onto PVDF membranes (Millipore Corporation, Billerica, MA, USA). After blocking with 5% skimmed milk powder in TBS-T for 2 h, the membranes were incubated overnight at 4 °C with the primary antibodies to NLRP3 (Cat. #ab263899; Abcam, Cambridge, MA, USA), the P2X7 receptor (Cat. #sc-514962; Santa Cruz Biotechnology, Inc., Santa Cruz, CA, USA), cleaved N-terminal GSDMD (Cat. #ab255603; Abcam, Cambridge, MA, USA), and cleaved-caspase-1 (Cat. #89332; Cell Signaling Technology, Danvers, MA, USA), and β-actin was used as an internal control. The membranes were washed and then incubated with secondary antibodies against horseradish peroxidase-conjugated rabbit or mouse IgG at room temperature. Enhanced chemiluminescence (CWBIO, Beijing, China) was used to visualize the immunoreactivity bands, which were captured through the ChemiDoc-IRT 510 image system (Upland, CA, USA). Protein expression levels were quantitatively analyzed by a Gel-Pro v. 4.0 analyzer (Media Cybernetics, Inc., Rockville, MD, USA).

### 4.11. Molecular Docking of Puerarin and the P2X7 Receptor Based on CDOCKER

We downloaded the mol2 format file of the puerarin structure from the TCMSP platform, and we downloaded the PDB format file of the target protein from the protein crystal structure database (http://www.rcsb.org, accessed on 4 July 2022). Molecular docking was performed using DS BIOVIA Discovery Studio Client 2016 v16.1 software, and the top hit was set to 10, random conformations were set to 10, orientations to refine were set to 10, and other parameters were set to default values.

CDOCKER mainly calculates the total energy CE (CDOCKER_ENERGY) and the receptor–ligand interaction energy CIE (CDOCKER_INTERACTION_ENERGY) of receptors and ligands as the scoring function. The lower the CE, the lower the total energy of the receptor and the ligand, indicating that the docking system is more stable. The lower the CIE, the smaller the interaction during the docking process, indicating that the ligand binds to the receptor better. However, in some cases, the docked small molecules are relatively rigid and have high self-energy, which is prone to inaccurate CE. Therefore, this study uses CIE as the scoring function for statistics.

### 4.12. Statistical Analysis

Data were expressed as the mean ± standard error of the mean (SEM) and analyzed using GraphPad Prism 8 (GraphPad Software, Inc., San Diego, CA, USA). Statistical analysis was performed using one-way analysis of variance (ANOVA) accompanied by Dunnett’s multiple comparison test. *p* < 0.05 was considered statistically significant.

## 5. Conclusions

**Figure 7:**
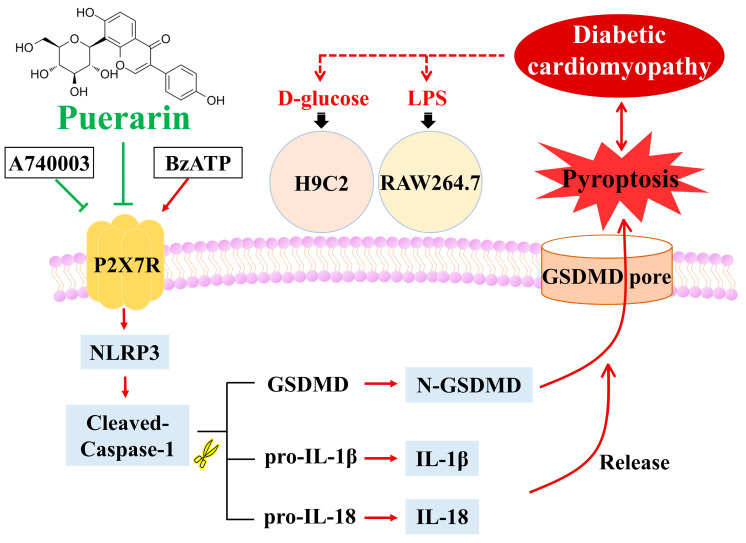
Schematic diagram of possible signal pathways of puerarin in D-glucose-induced H9C2 cells and LPS-induced RAW264.7 cells.

In conclusion, LPS-induced inflammation and hyperglycemia-induced cardiomyocyte dysfunction may promote the occurrence of pyroptosis. Our results indicate that D-glucose induced significant H9C2 cell death, and this is substantially reduced by puerarin treatment in a concentration-dependent manner in vitro. We proved that puerarin can improve cardiomyocytes’ mitochondrial respiratory dysfunction caused by high glucose using Oxygraph-2k high-resolution respirometry. We also explored the effect of puerarin on the inhibition of inflammation in RAW264.7 macrophages. Puerarin reduced the generation of inflammatory cytokines. Based on the results of in vitro experiments and molecular docking technologies, we were able to predict a role for puerarin in the regulation of the pyroptosis signaling pathways during diabetic cardiomyopathy, and this regulation was associated with the P2X7 receptor. A simplified overview of the above signaling pathways is illustrated in Figure 7. These data support that P2X7 receptor-mediated pyroptosis may be a key pathway for the treatment of diabetic cardiomyopathy with puerarin, which further corroborates the results of earlier animal experiments. These results may aid in the design and development of natural anti-inflammatory drugs for DCM.

## Figures and Tables

**Table 1:** The interaction binding energy of puerarin and the P2X7 receptor.

Protein	Activate Site	Ligand	-Cdocker Interaction Energy
P2X7 receptor	Activate site 1	Puerarin	29.205
		Original ligand 1	25.6637
P2X7 receptor	Activate site 2	Puerarin	27.435
		Original ligand 2	9.77534
P2X7 receptor	Activate site 3	Puerarin	18.8467
		Original ligand 3	9.39733
P2X7 receptor	Activate site 4	Puerarin	12.884
		Original ligand 4	33.258

**Table 2:** Primers for H9C2 and RAW264.7 cells.

Cell	Primer		Sequence
H9C2	IL-1β	F	CACCTCTCAAGCAGAGCACAG
		R	GGGTTCCATGGTGAAGTCAAC
	IL-18	F	TGGAGACTTGGAATCAGACC
		R	GGCAAGCTAGAAAGTGTCCT
	TNF-α	F	ACGTCGTAGCAAACCACCAA
		R	GCAGCCTTGTCCCTTGAAGA
	GAPDH	F	ATGGCACAGTCAAGGCTGAGA
		R	CGCTCCTGGAAGATGGTGAT
RAW264.7	NLRP3	F	AGCCTTCCAGGATCCTCTTC
		R	CTTGGGCAGCAGTTTCTTTC
	IL-1β	F	ATCTCGCAGCAGCACATCAA
		R	ATGGGAACGTCACACACCAG
	IL-18	F	AAAGAAAGCCGCCTCAAACCT
		R	AATCATCTTTCTGGAACACAA
	β-actin	F	TCTGTGTGGATTGGTGGCTCTA
		R	CTGCTTGCTGATCCACATCTG

## Data Availability

All data generated or analyzed during this study are included in this article and are available from the corresponding author upon reasonable request.
